# Supplementary material for: Insights into the structure and initial host attachment of the flagellotropic bacteriophage 7-7-1
Source: Commun Biol. 2025 Dec 6;9:55. doi: 10.1038/s42003-025-09319-7 (PMC12796354; doi:10.1038/s42003-025-09319-7)
Supplement: Supplementary file 1 — Supplementary material [file 42003_2025_9319_MOESM1_ESM.pdf]

Supplementary Materials

Insights into the structure and initial host attachment of the flagellotropic bacteriophage 7-7-1

Noteborn, W.E.M., Ouyang, R., Hoeksma, T., Sidi Mabrouk, A., Esteves N.C., Pelt, D.M., Scharf, B.E., Briegel, A.

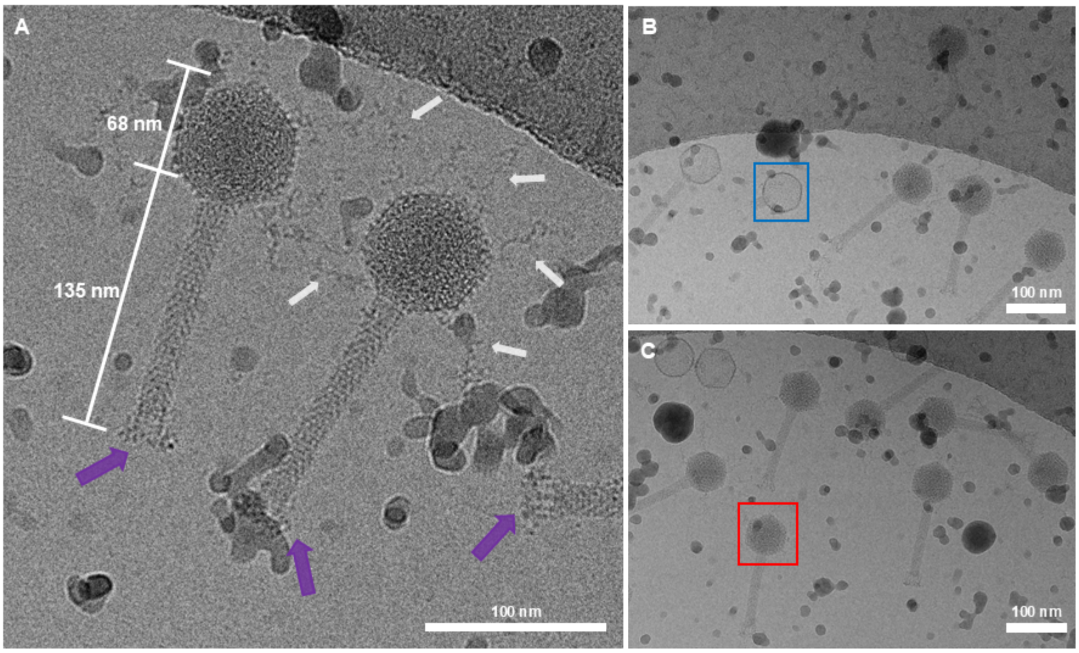

Supplementary Figure 1 | Representative micrographs of phage 7-7-1. (A) An enlarged image of a representative cryo-EM image from SPA. This image showcases a hexagonal head with a diameter around 68 nm and a contractile tail measuring 135 nm in length. Notably, the end of the contractile tail is decorated with abundant bushy tail fibers (purple arrows), and many long-curved capsid fibers surrounding the capsid (white arrows). **(B)** A representative micrograph of phage 7-7-1 with empty capsids (blue rectangle). **(C)** A representative micrograph of phage 7-7-1 with full capsids containing DNA (red rectangle). Notably, most of the phages observed in our dataset were intact capsids filled with DNA. All scale bars represent 100 nm.

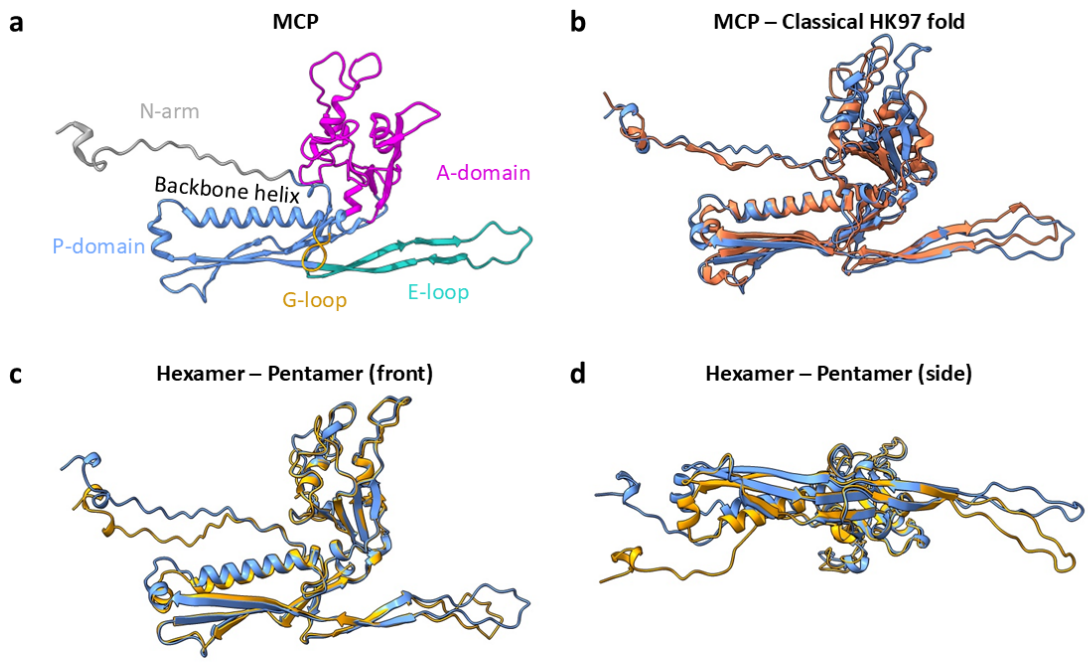

Supplementary Figure 2 | Structural comparison of MCP with the classical HK97 fold and differences of the MCP in the hexamer and pentamer conformation. The MCP consists of the classical HK97 domains **(a)** and bears structural resemblance to the MCP of phage HK97 (PDB: 1OHG) The RMSD is 1.3Å in the highly conserved regions and 6.8 Å overall. The MCP studied here is colored blue and the HK97 MCP is colored orange (**b**). The differences between the hexamer and pentamer MCP conformation as subtle, but are most dominant in the N-arm and E-loop: front view **(c)**, side view **(d)**.

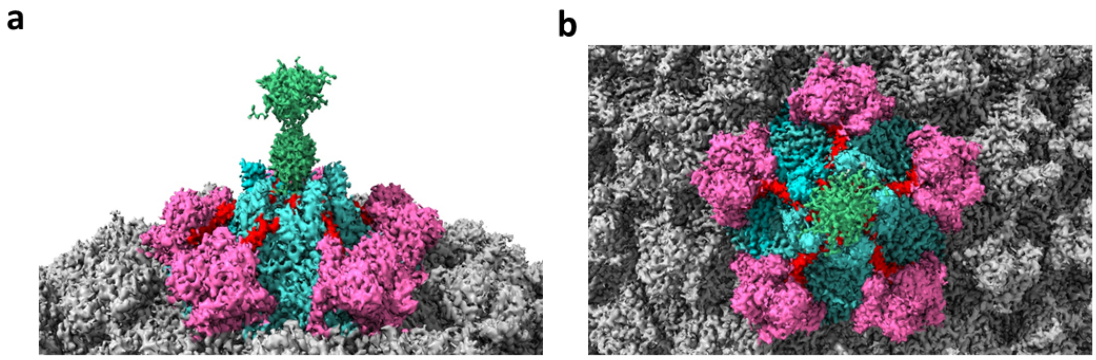

Supplementary Figure 3 | Zoomed-in visualization of the poorly resolved Ig-type domain of LP2 that protrudes the pentamer into the capsid fiber. A side-view is shown in (a) and a top-view in (b).

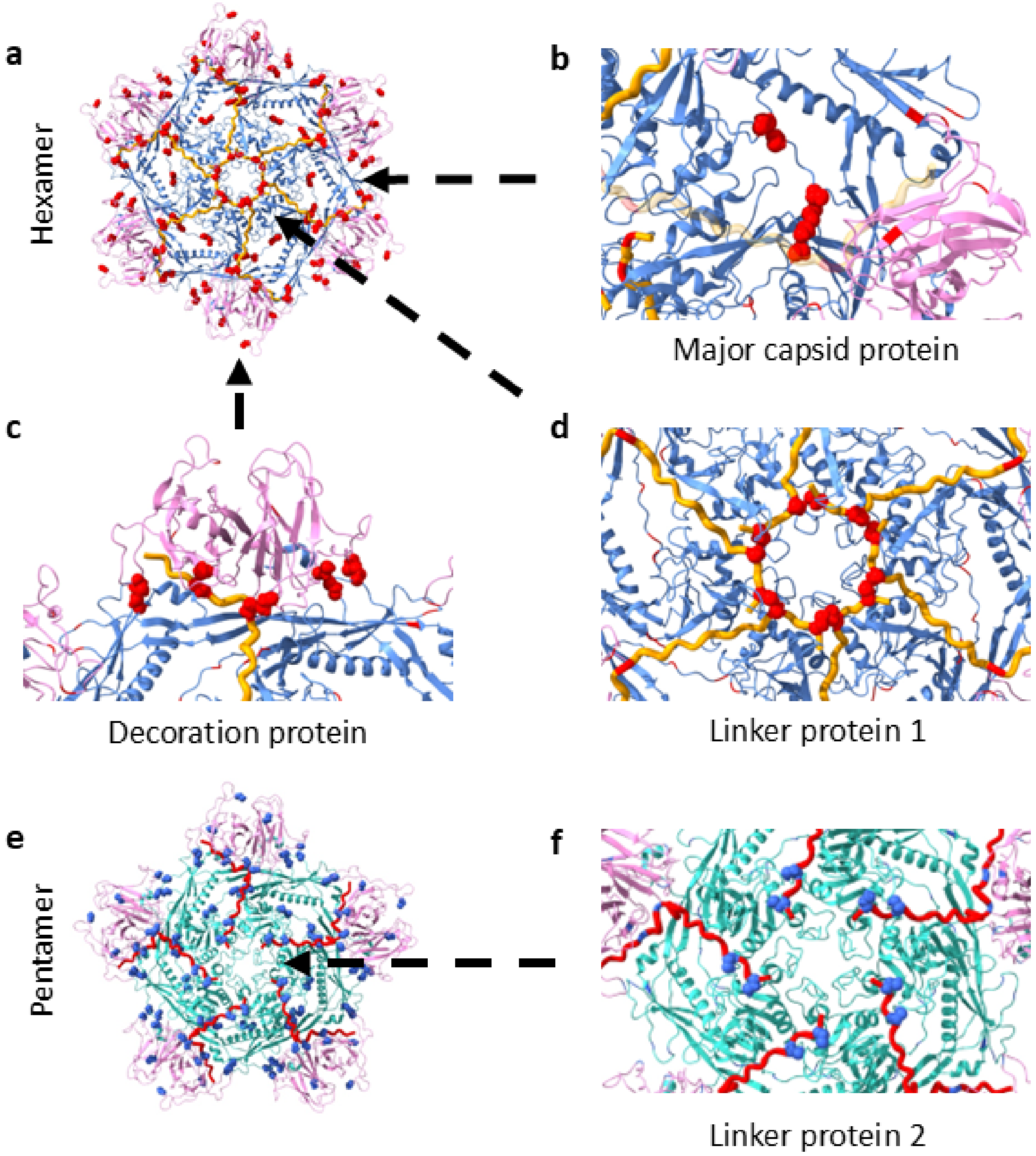

Supplementary Figure 4 | Overview of the different disulfide bonds present in the hexameric and pentameric assembly units. The hexamer unit **(a)** harbors 54 disulfide bonds (highlighted in red). They can be distinguished in three separate groups: disulfide bonds in the MCP **(b)**, DP **(c)**, and LP1 **(d)**. The pentamer **(e)** harbors 35 disulfide bonds (highlighted in blue) and is largely identical to the hexameric conformation, only with LP2 forming one less disulfide bond with DP and no disulfide bonds between the adjacent LP2 monomers **(f)**.

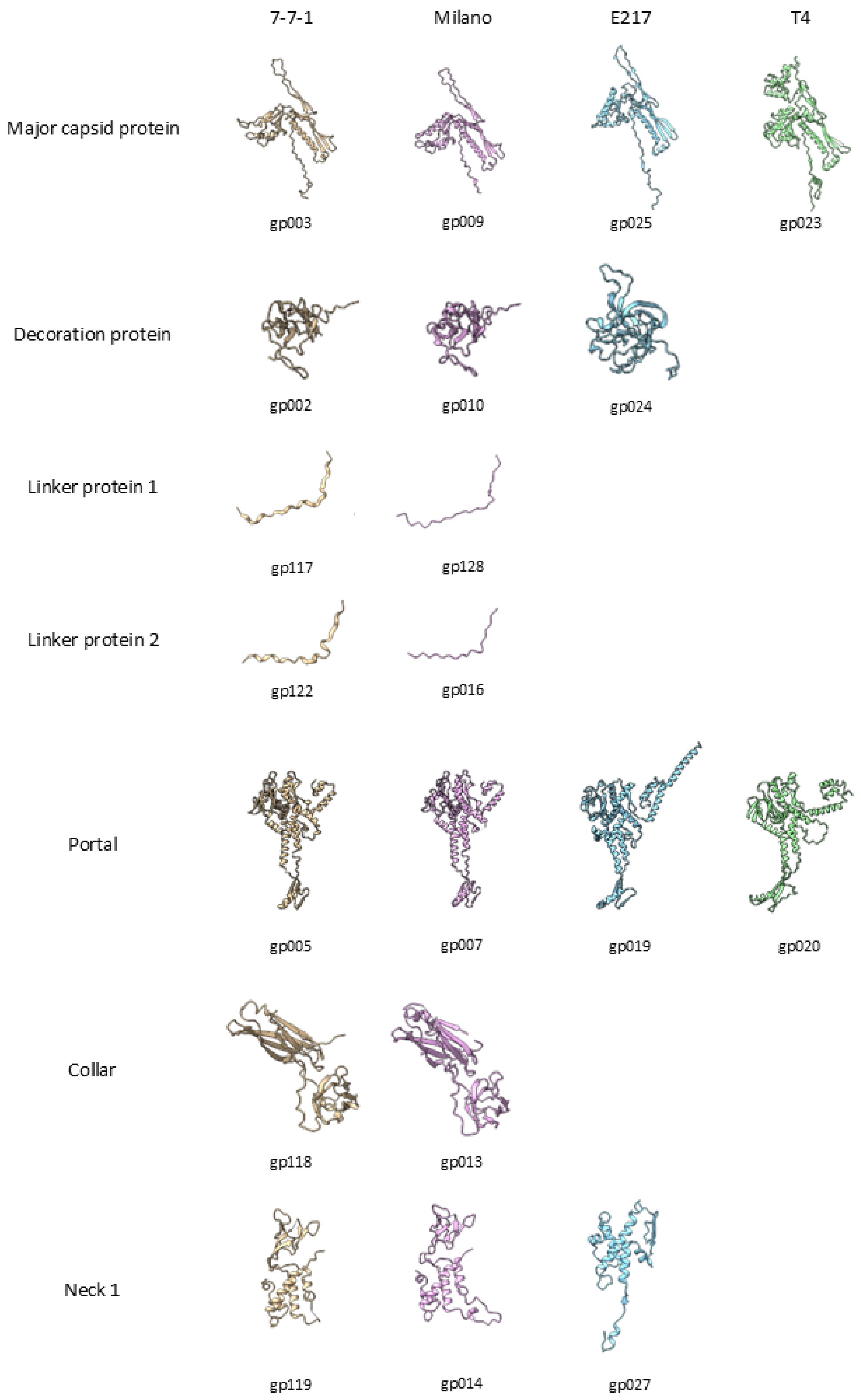

Supplementary Figure 5 | Comparison of phage 7-7-1 structural proteins with phages Milano, E217 and T4 (part 1/3). Reference PDB structures used to make the comparisons: Milano capsid: 8FXP, Milano neck region: 8FWE, Milano tail region: 8FOP, Milano baseplate: 8FQC. E217 capsid: 8FRS, E217 neck region: 8FVH, E217 tail region: 8FUV, E217 baseplate: 8EON, T4 capsid: 5VF3, T4 portal: 3JA7, T4 tail region: 3J2M, T4 baseplate: 5IV5.

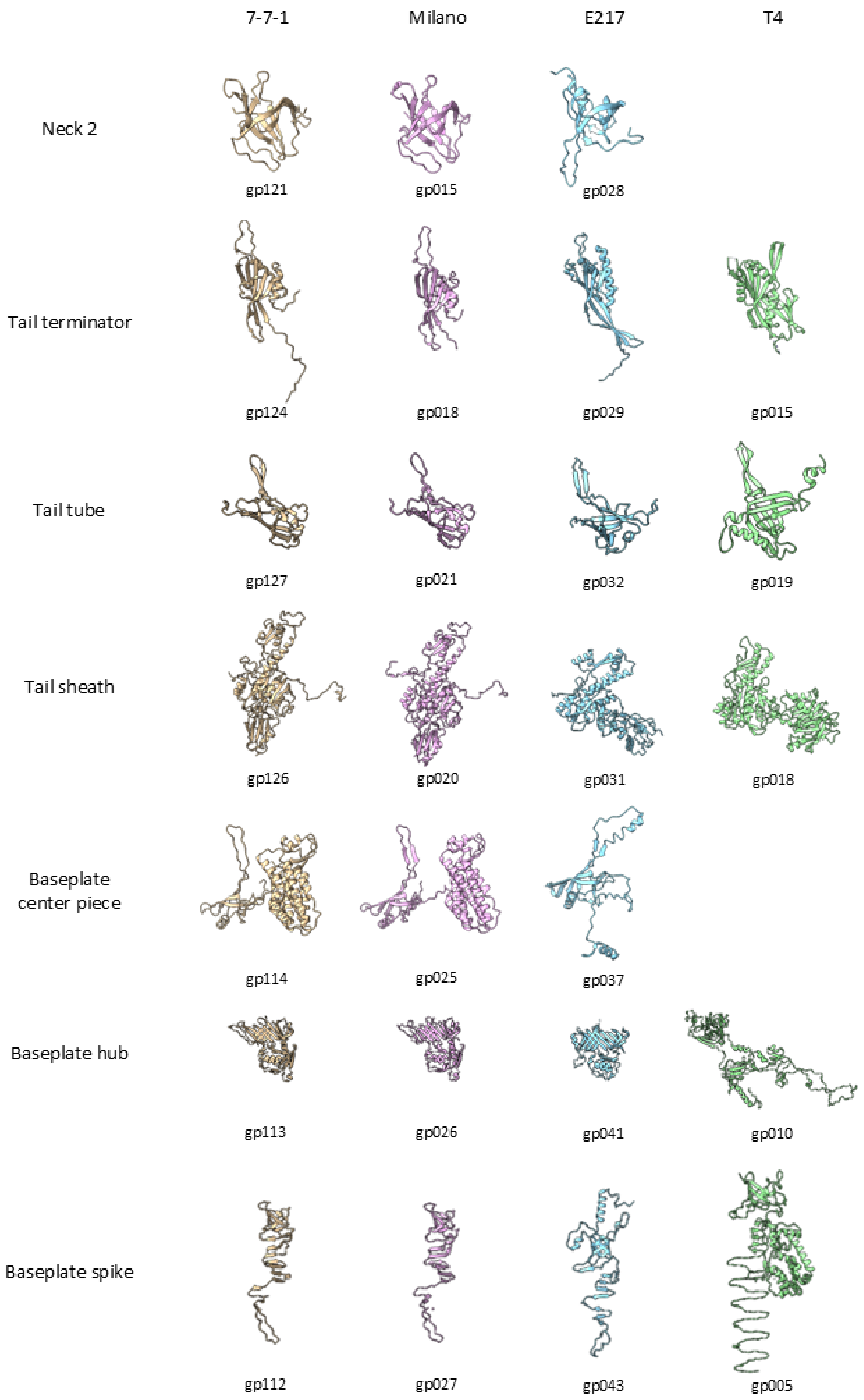

Supplementary Figure 5 | Comparison of phage 7-7-1 structural proteins with phages Milano, E217 and T4 (part 2/3).

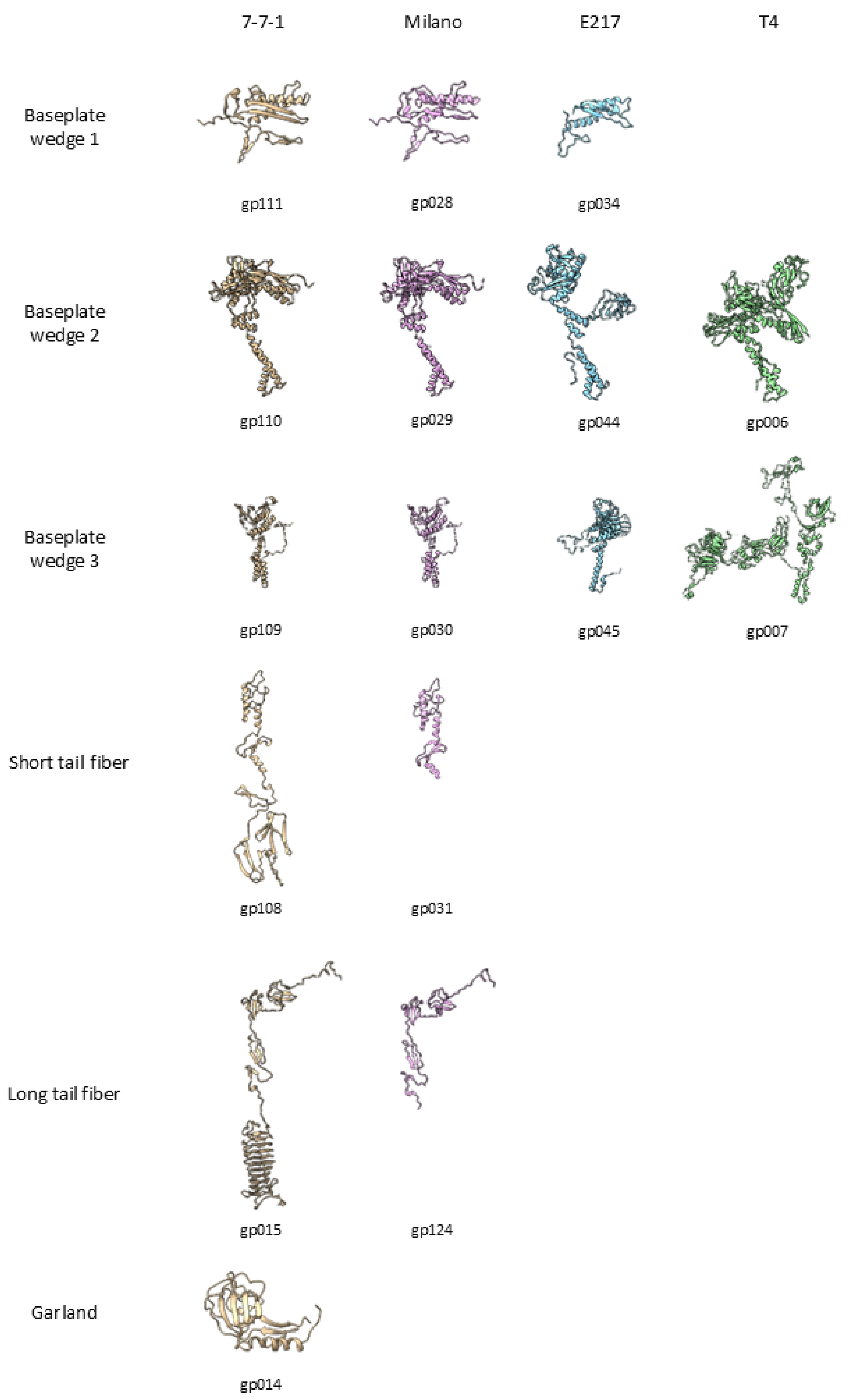

Supplementary Figure 5 | Comparison of phage 7-7-1 structural proteins with phages Milano, E217 and T4 (part 3/3).

Supplementary Table 1 | Comparison of phage 7-7-1 structural proteins with phages Milano, E217 and T4 by RMSD and TM-score.

	7-7-1 vs Milano		7-7-1 vs E217		7-7-1 vs T4	
	RMSD	TM-score	RMSD	TM-score	RMSD	TM-score
**Major capsid protein**	1.3 (290/305)	0.92	4.0 (271/305)	0.71	3.7 (264/305)	0.69
**Decoration protein**	1.2 (135/135)	0.95	5.5 (72/135)	0.26	---	---
**Linker protein 1**	1.4 (26/27)	0.45	---	---	---	---
**Linker protein 2**	1.8 (19/27)	0.28	---	---	---	---
**Portal**	1.4 (376/390)	0.93	4.7 (330/390)	0.64	5.0 (306/390)	0.59
**Collar**	1.1 (207/208)	0.96	---	---	---	---
**Neck 1**	1.0 (161/164)	0.94	3.4 (92/164)	0.42	---	---
**Neck 2**	0.9 (125/126)	0.95	3.7 (102/126)	0.56	---	---
**Tail terminator**	1.3 (153/173)	0.84	4.2 (140/173)	0.58	4.0 (131/173)	0.56
**Tail tube**	0.7 (131/133)	0.96	3.3 (121/133)	0.64	3.2 (111/133)	0.58
**Tail sheath**	2.4 (490/500)	0.93	4.5 (337/500)	0.54	4.9 (301/500)	0.47
**Baseplate center piece**	0.8 (394/398)	0.97	3.5 (116/398)	0.25	---	---
**Baseplate hub**	1.0 (324/325)	0.97	3.3 (241/325)	0.62	3.5 (295/325)	0.74
**Baseplate spike**	1.0 (168/172)	0.94	4.1 (132/172)	0.53	4.4 (105/172)	0.54
**Baseplate wedge 1**	1.1 (175/175)	0.96	2.8 (105/175)	0.46	---	---
**Baseplate wedge 2**	0.7 (390/391)	0.99	5.3 (128/391)	0.34	5.5 (184/391)	0.57
**Baseplate wedge 3**	0.6 (285/286)	0.99	4.6 (124/286)	0.41	5.8 (87/286)	0.33
**Short tail fiber**	1.0 (119/297)	0.95 *	---	---	---	---
**Long tail fiber**	1.6 (173/560)	0.92 *	---	---	---	---
**Garland**	---	---	---	---	---	---

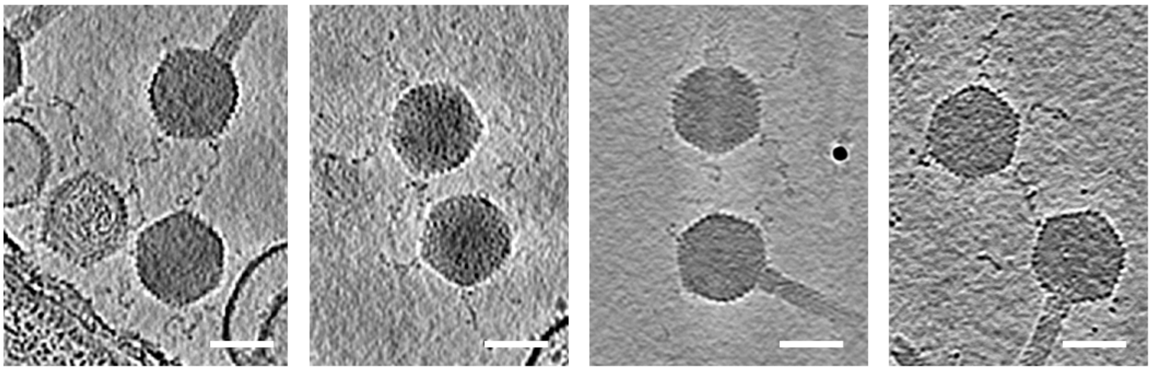

Supplementary Figure 6 | Interactions between the capsid fibers of different phages. Additional examples of interacting capsid fibers (scale bar: 50 nm).

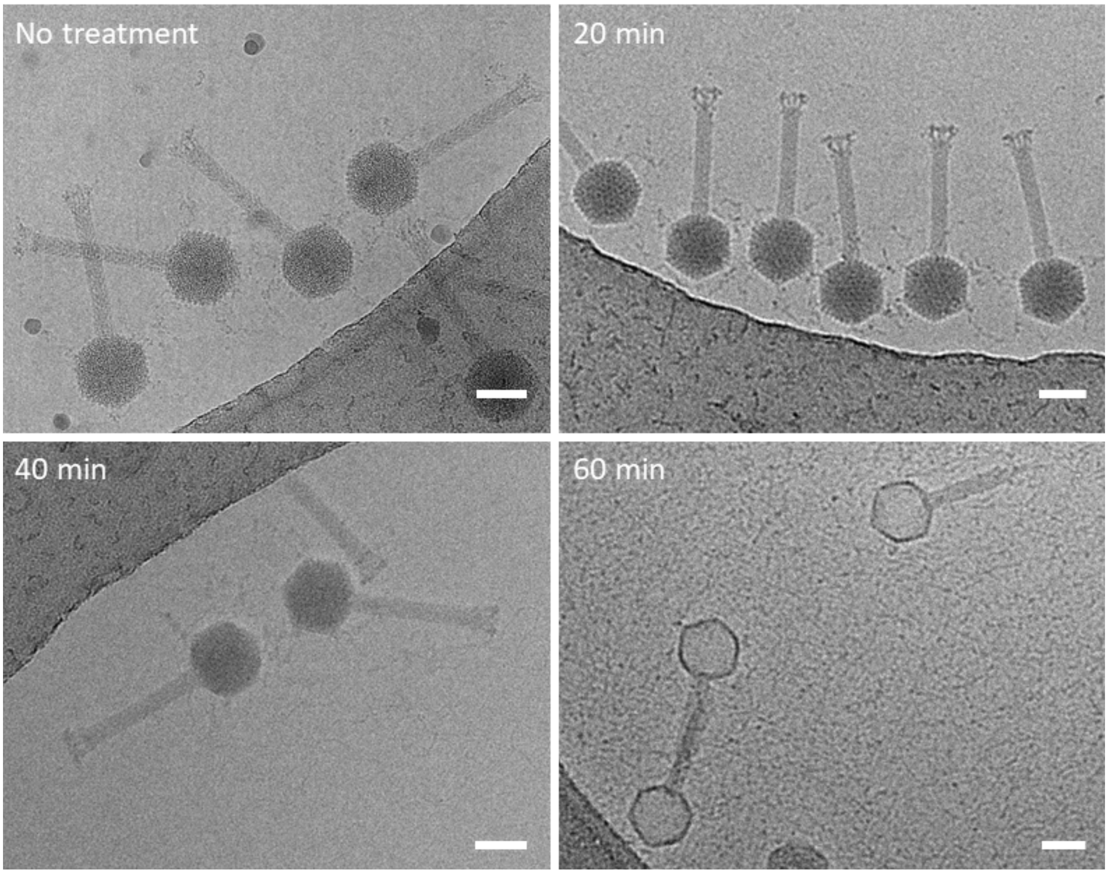

Supplementary Figure 7 | Effect of Proteinase K degradation on capsid fiber over time. Although Proteinase K is not selective towards specific proteins, these representative cryo-EM micrographs show the degradation of the capsid and the capsid fibers of bacteriophage 7-7-1 in comparison of a no-treatment control (top left), after 20 minutes (top right), 40 minutes (bottom left), and 60 minutes (bottom right) of Proteinase K treatment. Scale bars are 50 nm. Red arrows highlight the capsid fibers, the white arrow highlights a small part of remaining capsid fiber after 60 minutes of treatment.

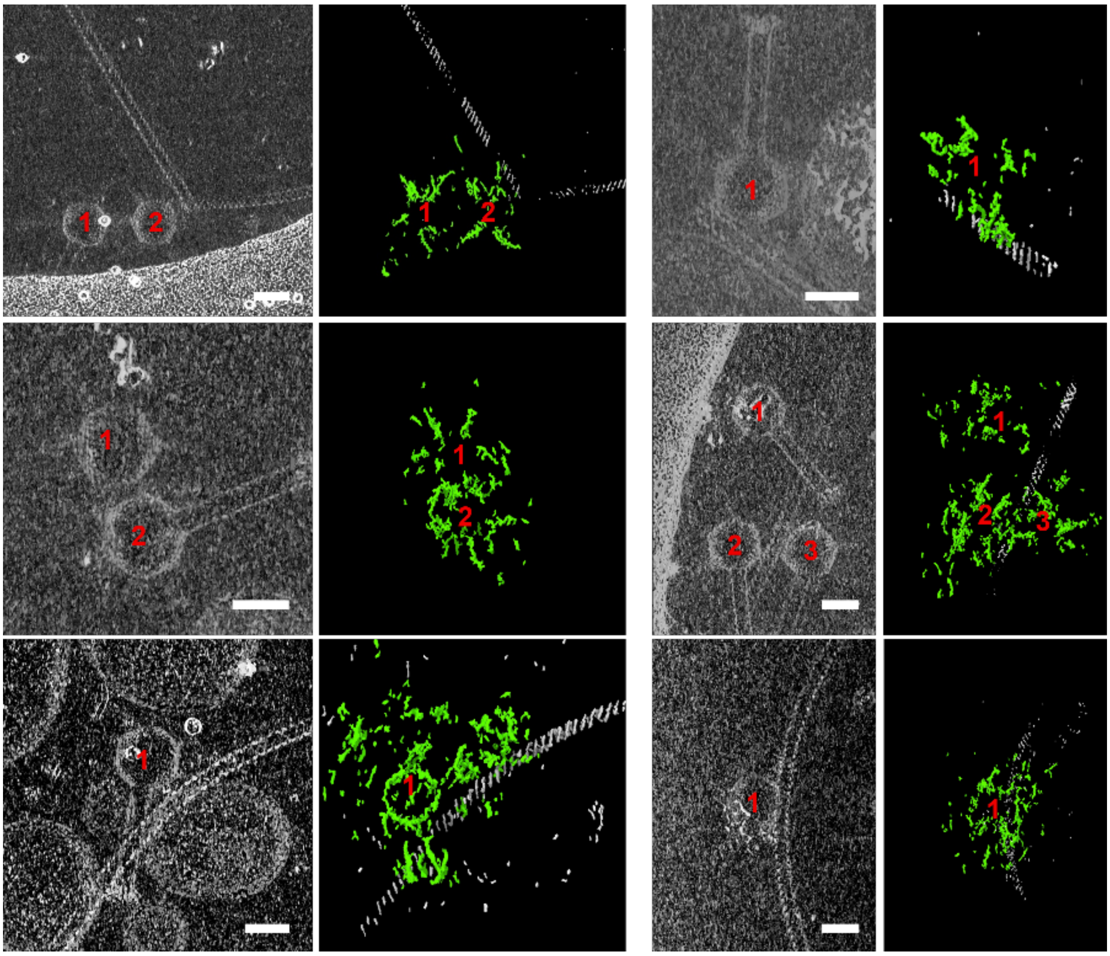

Supplementary Figure 8 | Trimmed tomograms of phage 7-7-1 attached to flagella and 3D structures of capsid fibers. Six groups of trimmed tomograms and their corresponding 3D structures. In each group, the left figure displays a trimmed tomogram, which is overlaid and visualized into 2D image, created through the 3D projection function in FIJI. On the right, the 3D structures of capsid fibers (green) and flagella (gray) are visualized, generated using the neural network. Red numbers are marked at the center of phage capsids for location and identification purposes. Scale bars: 50 nm.

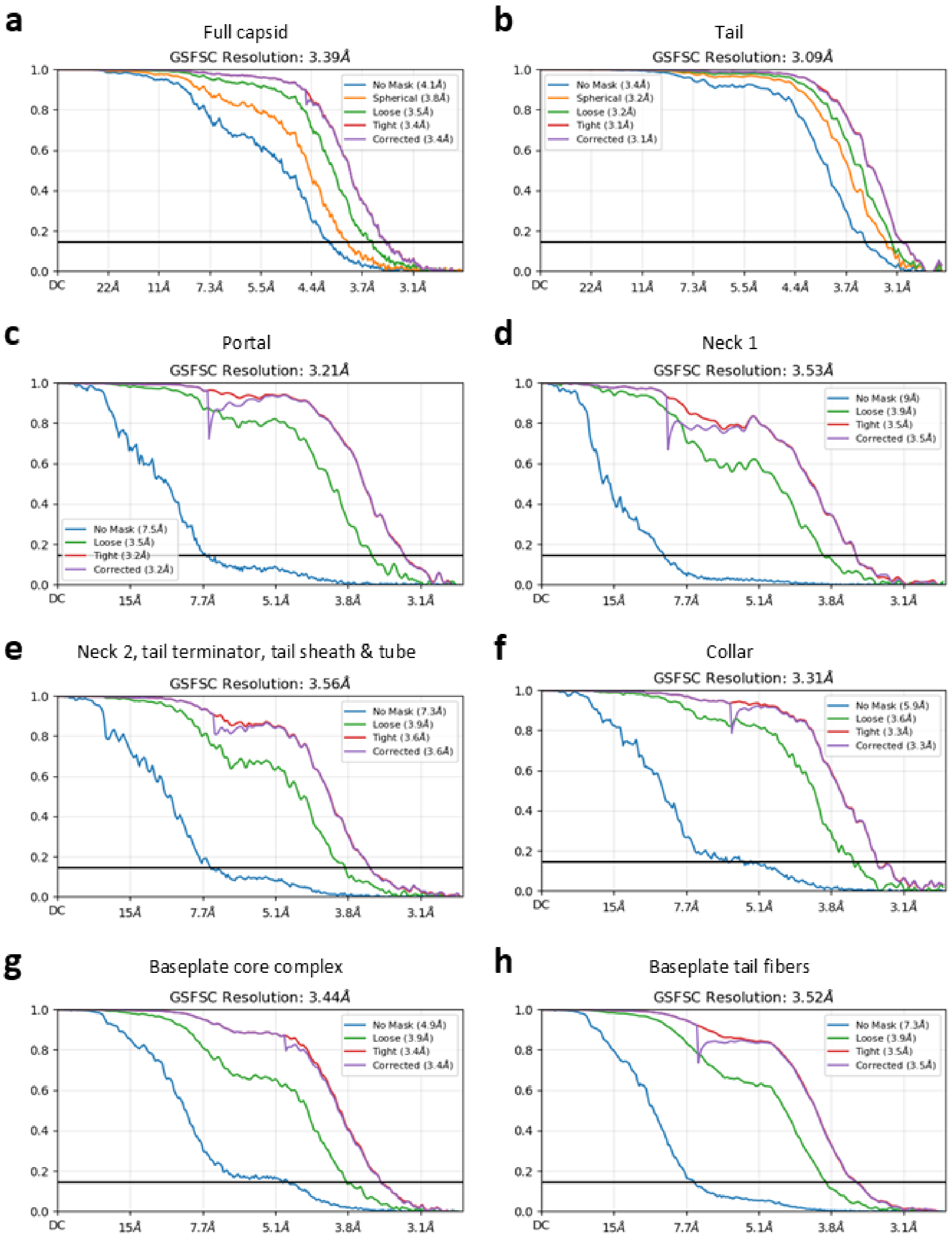

Supplementary Figure 9 | Fourier shell correlation curves. Map-map FSC curves of the phage 7-7-1 (a) capsid, (b) tail, (c) portal, (d) neck 1, (e) neck 2, tail terminator, tail sheath and tail tube, (f) collar, (g) baseplate core complex, and (h) baseplate tail fibers at a cut-off of 0.143.

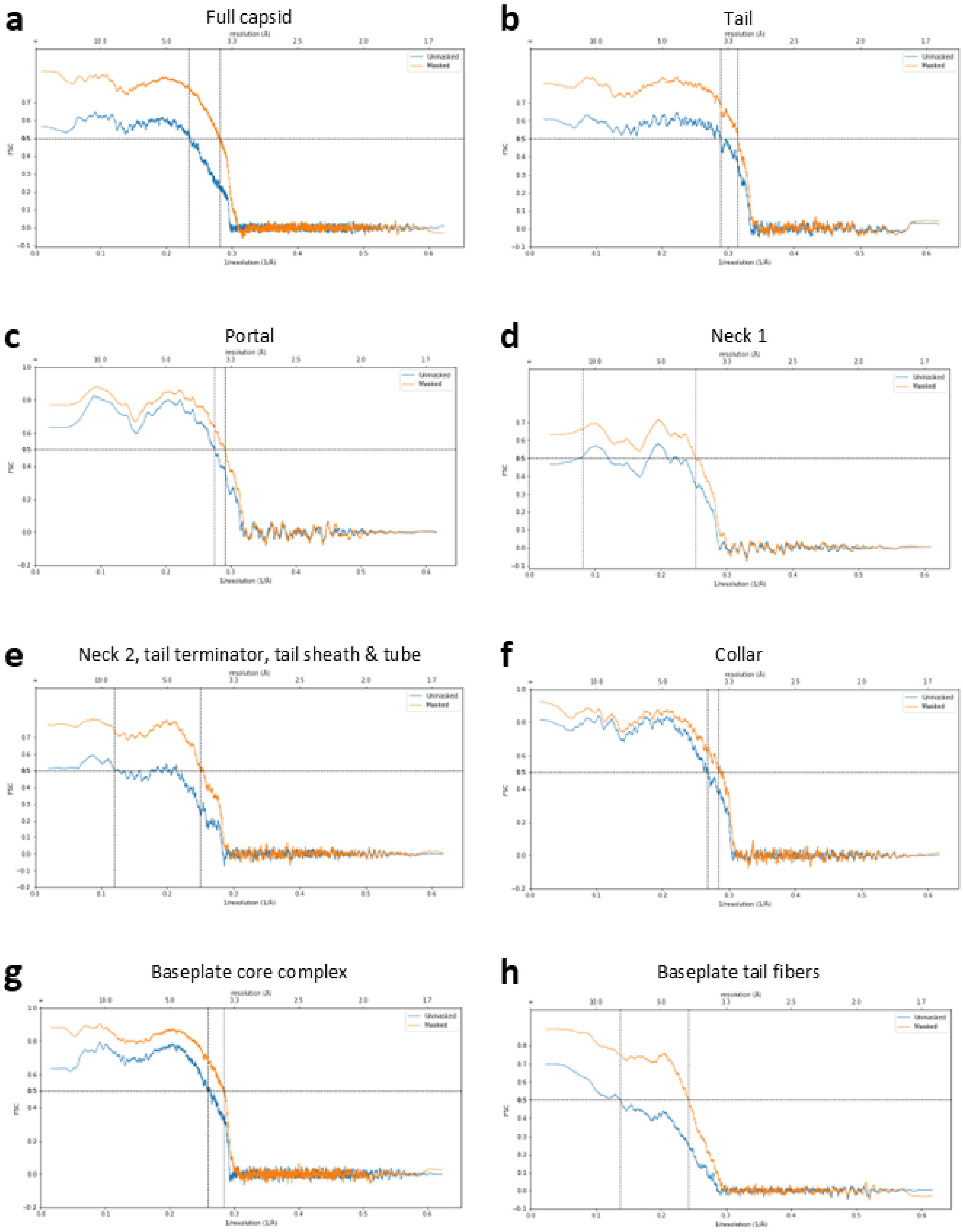

Supplementary Figure 10 | Fourier shell correlation curves. Map-model FSC curves of the phage 7-7-1 (a) capsid, (b) tail, (c) portal, (d) neck 1, (e) neck 2, tail terminator, tail sheath and tail tube, (f) collar, (g) baseplate core complex, and (h) baseplate tail fibers at a cut-off of 0.5.
