# Supplementary material for: Insights into the structure and initial host attachment of the flagellotropic bacteriophage 7-7-1
Source: Commun Biol. 2025 Dec 6;9:55. doi: 10.1038/s42003-025-09319-7 (PMC12796354; doi:10.1038/s42003-025-09319-7)
Supplement: Supplementary file 2 — Description of Additional Supplementary Files [file 42003_2025_9319_MOESM2_ESM.docx]

**Description of Additional Supplementary Files

File name:** Supplementary Data **Description:** Source data for Supplementary Figures 9 and 10
